# Prevalence of Erectile Dysfunction and Help-Seeking Behavior Among Patients Attending Primary Healthcare Centers for Non-Urological Complaints

**DOI:** 10.3390/healthcare13091088

**Published:** 2025-05-07

**Authors:** Mansour Alnazari, Sulaiman Abdullah, Abdullah K. Aljohani, Emad S. Rajih, Ghadi S. Alghamdi, Faris S. Sebaa, Ali A. Alraddadi, Wesam Khan, Adel Moalwi

**Affiliations:** 1 Department of General and Specialized Surgery, College of Medicine, Taibah University, Madinah 42361, Saudi Arabia; remad@taibahu.edu.sa; 2 College of Medicine, Taibah University, Madinah 42361, Saudi Arabia; tu4101747@taibahu.edu.sa (S.A.); abdullahas535@gmail.com (A.K.A.); 3 College of Medicine, Al-Baha University, Albaha 61008, Saudi Arabia; d.ghadi6@hotmail.com; 4 Alrayan College of Medicine, Madinah 42541, Saudi Arabia; sebaa.fff@gmail.com (F.S.S.); alirddadi@gmail.com (A.A.A.); 5 Department of Surgery, Faculty of Medicine, University of Tabuk, Tabuk 71491, Saudi Arabia; wkhan@ut.edu.sa; 6 Department of Surgery, College of Medicine, Najran University, Najran 66462, Saudi Arabia; aaassiry@nu.edu.sa

**Keywords:** erectile dysfunction, help-seeking behavior, Saudi Arabia

## Abstract

**Background/Objectives**: Erectile dysfunction (ED) is a prevalent condition worldwide that significantly affects men’s sexual health and overall quality of life. ED is often associated with both psychological and organic factors and may serve as an early indicator of underlying health conditions such as diabetes mellitus, hypertension, and cardiovascular diseases. This study aimed to assess the attitudes and help-seeking behaviors of patients with ED who attended primary healthcare centers for non-urological reasons. **Methods**: A cross-sectional survey was conducted among 384 men aged 18 years and older who attended primary care clinics. Erectile dysfunction was evaluated using a structured questionnaire designed to assess patients’ attitudes toward ED, help-seeking behaviors, and treatment preferences. **Results**: Approximately half of the participants (49.5%) acknowledged the necessity of seeking treatment. However, the majority (53.1%) had not consulted specialized clinics, primarily due to social stigma and a preference for self-medication. Concerning sources of information on ED treatment, 30.7% of participants relied on their partners and healthcare providers. **Conclusions**: The study underscores critical barriers to ED management, including social stigma and reliance on self-medication, which may impede optimal treatment engagement and access to specialized care.

## 1. Introduction

Erectile dysfunction (ED) is a prevalent andrological condition that significantly impacts sexual health and quality of life [1]. It manifests as difficulty in achieving or maintaining an erection sufficient for sexual activity [2]. ED is a multifactorial condition resulting from complex interactions among biological, psychological, and social factors, such as vascular or neurological impairments, hormonal imbalances, performance anxiety, and tobacco use [3]. Consequently, ED serves as a potential indicator of underlying systemic diseases, such as diabetes mellitus, hypertension, and cardiovascular diseases [4]. Identifying the underlying cause of ED is essential for effective treatment, as it enables the management of both medical and psychosocial contributors [5].

Despite its high prevalence, a substantial proportion of individuals with ED do not seek medical care, and many healthcare providers hesitate to initiate discussions about sexual health due to cultural and social barriers. This lack of communication reduces quality of life and delays the diagnosis of more serious conditions that could contribute to increased morbidity and mortality [6]. Estimating the exact prevalence of ED is challenging, as it varies widely from 2% to 80% worldwide and has been reported at 10.53% among married men in Saudi Arabia [7,8]. Exploring patients’ attitudes and help-seeking behaviors is key to developing tailored treatment strategies that reflect the complex nature of ED [9].

Several studies suggest that phosphodiesterase type 5 inhibitors (PDE5Is) significantly improve erectile function. However, 20–30% of men experience unsatisfactory outcomes with oral PDE5Is [10]. While the majority of patients achieve satisfactory results, 31–57% discontinue treatment, often due to factors such as low educational levels and insufficient knowledge about ED and its management [11]. In addition, alternative treatment modalities such as intracavernosal injections, vacuum erection devices, and low-intensity shockwave therapy are also available, particularly for patients who are non-responsive to PDE5Is or for whom such medications are contraindicated.

The present study aimed to assess the attitudes of patients with ED toward their condition and available treatment options, as well as to investigate their help-seeking behaviors among individuals attending primary healthcare centers for non-urological complaint in Saudi Arabia.

## 2. Materials and Methods

### 2.1. Study Design and Setting

This cross-sectional study was conducted at public primary healthcare centers in Madinah, Saudi Arabia. The study aimed to assess the prevalence of ED and the associated help-seeking behaviors among men aged 18 years and older.

### 2.2. Study Population

The study included 384 men who visited primary care clinics. Participants were eligible if they were aged 18 years or older, attended the clinics during the study period from October to December 2023, and provided informed consent to participate.

### 2.3. Data Collection

Data were collected through a structured questionnaire, which was developed based on the study objectives and reviewed by experts in the field to ensure content validity. The questionnaire comprised sections on sociodemographic characteristics, lifestyle factors, medical history, and a validated Arabic version of the International Index of Erectile Function (IIEF-15); this version has been previously utilized in studies conducted among Saudi male populations [7,9]. The IIEF-15 assesses five domains: erectile function, orgasmic function, sexual desire, intercourse satisfaction, and overall satisfaction. Scores for each domain range from 0–5 or 1–5, with higher scores indicating better function. Sociodemographic data included age, education level, and monthly income. Lifestyle factors encompassed physical activity levels and smoking status. Medical history focused on common chronic conditions, including diabetes mellitus, hypertension, and hypercholesterolemia.

### 2.4. Statistical Analysis

Data were analyzed using IBM SPSS Statistics, version 26. Associations between erectile dysfunction and potential predictors were examined using the Chi-square test for categorical variables. Descriptive statistics were presented as percentages and frequencies. Statistical significance was defined as a *p*-value < 0.05 for all tests.

### 2.5. Ethical Considerations

Ethical approval for the study was obtained from the Institutional Review Board of General Directorate of Health Affairs in Madinah National Registration Number with NCBE-KACST, KSA: (H-03-M-84), IRB log No: 23-090 on 21 September 2023. Written informed consent was obtained from all participants before enrollment. Confidentiality and anonymity of participant data were strictly maintained throughout the study, with no personal identifiers recorded or reported.

## 3. Results

### 3.1. Study Population

A total of 384 men aged 18 years and older who attended primary healthcare centers in Madinah, Saudi Arabia, were enrolled in the study. The mean age of participants was 39 ± 8.5 years. A detailed breakdown of sociodemographic characteristics is provided in Table 1.

### 3.2. Prevalence of Erectile Dysfunction

**Figure 1:**
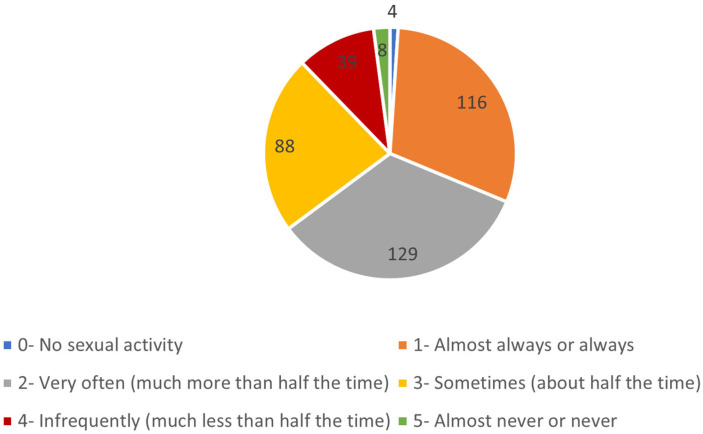
Distribution of participants based on their ability to achieve an erection during sexual activity in the past four weeks. This figure illustrates the frequency with which participants reported being able to achieve an erection during sexual activity over the past month.

As illustrated in Figure 1, 30.2% of participants reported that they were almost always able to achieve an erection, whereas 2.1% reported that they were almost never able to do so. Table 2 presents detailed responses regarding the ability to maintain an erection sufficient for penetration and during intercourse. Notably, 32.3% of participants reported always or almost always having an erection sufficient for penetration.

### 3.3. Help-Seeking Behaviors and Treatment Outcomes

**Figure 2:**
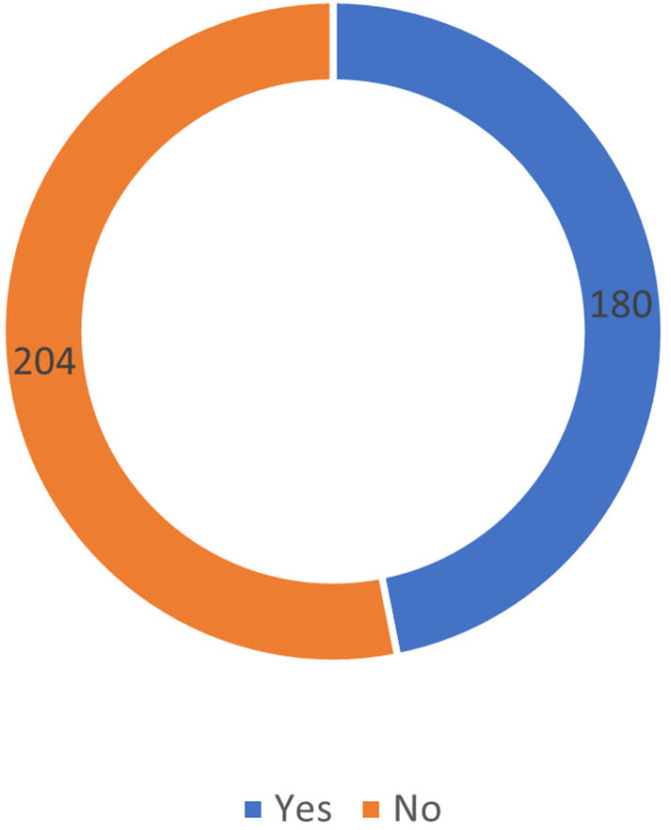
Proportion of participants who consulted male clinics regarding ED. This figure shows the percentage of participants who sought consultation at specialized male clinics for erectile dysfunction and the percentage who did not.

Despite the prevalence of ED symptoms, only 46.9% of participants had consulted a healthcare professional regarding their erectile difficulties (Figure 2). As reported in Table 3, the most common treatment modality was medical treatment (37.4%), followed by alternative approaches (34.3%) and self-directed methods (28.3%). Half of the study population (50.5%) perceived no need for treatment.

### 3.4. Associations with Sociodemographic and Lifestyle Factors

Chi-square analysis identified a significant association between age and medication use for ED (*p* = 0.019), with older participants more likely to use medication (Table 4). However, no significant associations were found between healthcare consultation and various sociodemographic factors (Table 5).

### 3.5. Knowledge and Awareness

A treatment uptake gap was observed, as only 47.1% of participants reported taking medication for ED. The primary sources of information about ED were doctors and spouses, each accounting for 30.7% of responses (Table 3). Concerns regarding the safety of sexual enhancers were prevalent, with 41.1% of participants perceiving them as unsafe due to potential adverse effects.

## 4. Discussion

Our findings underscore several critical aspects of ED among men in Madinah, Saudi Arabia, revealing a notable prevalence of ED and distinct variations in management and help-seeking behaviors. Approximately 30% of participants consistently achieved an erection sufficient for sexual activity; however, fewer than half sought professional consultation regarding their condition. It is important to recognize that ED prevalence varies by region and depends on the criteria and tools used for assessment. Although international studies report ED prevalence up to 52% in men aged 40–70 years [12,13], our findings showed lower rates of severe dysfunction. Only 2.1% of participants reported being almost never able to achieve an erection, while 10.2% did so infrequently. However, a large proportion reported partial difficulties (22.9% sometimes, and 33.6% very often), indicating that varying degrees of erectile issues are common. The inclusion of younger participants (28.4% under age 34) likely contributed to the lower rate of severe ED, yet 45.4% reported some level of difficulty maintaining erections, and only 11.7% expressed very high confidence. These findings highlight a significant burden of functional sexual concerns even in a relatively young population.

Erectile dysfunction is a multifactorial condition influenced by an intricate interplay of vascular, neurological, hormonal, and psychological factors. Vascular complications, particularly those arising from atherosclerosis and endothelial dysfunction, play a substantial role in the pathophysiology of ED and are frequently associated with systemic illnesses such as hypertension and diabetes [14]. Neurologically, conditions that impair nerve function or spinal integrity can disrupt the neural pathways required for normal erectile function [15]. In addition, testosterone levels significantly influence libido and erectile function, with hypogonadism being associated with a higher prevalence of ED [16]. Psychological factors such as depression, anxiety, and stress also contribute to both the onset and severity of ED, reinforcing its biopsychosocial nature [17]. The most common cause of ED, regardless of the age of the patient, is vasculogenic due to veno-occlusive dysfunction or venous leakage. However, the specific aging related ED is caused by gradual degradation and dysfunction of the corporal smooth muscle cells leading to the inability of the corporal tissue to prevent the blood from “leaking” out of the corporal sinusoids into the systemic veins. In addition, development of comorbidities such as hypertension and diabetes mellitus in aging males leads to various arterial diseases that contribute to ED [18]. We considered the need to take medication as a surrogate marker for the development of ED. In our study, increasing age was associated with the use of medication for ED. This observation was in agreement with previous reports from various parts of the world [19]. In another study in a Mediterranean country, the prevalence of severe ED increased from 2.7% in men in their twenties to 38.6% in their sixties and 46% in those aged 70 years and above. While age was the single most significant risk factor, other important risk factors included lower household income, physical inactivity, obesity, smoking, diabetes mellitus, hypertension, and ischemic heart disease [20]. In a previous study in Jeddah, Saudi Arabia, too, smoking was not considered as a risk factor for ED. However, patients with hypertension, diabetes mellitus, and ischemic heart disease had higher risk of developing a severe form of ED [21]. However, in our study, no statistically significant associations were found between ED and socio-demographic factors such as education level, number of wives, or chronic conditions like hypertension and diabetes mellitus, as detailed in Table 4 and Table 5 (*p* > 0.05). While these trends are noteworthy, no definitive conclusions can be drawn at this point. Further research with larger sample sizes and enhanced statistical power is necessary to verify or challenge these associations.

Although comorbidities such as diabetes, hypertension, and cardiovascular disease are well-established risk factors for erectile dysfunction [2,4], our findings showed that 28.2% of participants had no reported chronic medical conditions. Despite this, a considerable number within this subgroup reported varying degrees of erectile difficulty. This suggests that ED can occur even in the absence of organic disease and may reflect the influence of psychogenic or lifestyle-related factors. Psychological contributors such as anxiety, stress, and performance-related pressure have been recognized as key elements in younger men presenting with ED [8]. These findings emphasize the need for comprehensive assessment strategies that consider both organic and non-organic causes, even among seemingly healthy individuals.

By providing an expert opinion at any time, free of charge and in complete privacy, pharmacists play a pivotal part in promoting self-care interventions for sexual and reproductive health in the Eastern Mediterranean Region [22]. Among the participants in our study, 28% and 24% mentioned healthcare professionals including doctors and pharmacists and 21% mentioned wives as their major source of information. Earlier studies in western countries including the United Kingdom [23] and France [24] found that men sought help for sexual problems mostly from their sexual partners, followed by physicians. In more recent studies, the internet emerged as one of the most-consulted sources, and young patients tended to rely more on the internet [25,26]. In contrast, only about 15% of participants relied on social media as their internet-based source of information. The internet can be an important source of information for ED, especially for patients with a higher level of education and ability to search for and identify reliable content, whereas people with low health literacy may feel more comfortable navigating the readily accessible videos on social media such as YouTube. However, the scientific accuracy of information available on social media is often poor and untrustworthy. A recent study that assessed the scientific quality of Arabic-language video content related to erectile dysfunction highlighted that 84% of video content available on YouTube was not based on scientific evidence [27]. Therefore, for better health literacy around ED, it is not only important to popularize the use of the internet, but also to enable the users to access accurate content instead of relying on popularity-driven metrics.

Although the necessity of seeking treatment was acknowledged by half of the participants, the social stigma and a preference for self-medication inhibited half of the participants from reaching out to specialized clinics. The observed underutilization of ED healthcare services in our study is consistent with international evidence suggesting that stigma and cultural barriers significantly influence help-seeking behavior [28]. In terms of treatment approaches, medical therapy—mainly PDE5 inhibitors—was the most reported method, followed by using alternative and self-directed treatments. This trend reflects the findings of Albarakati et al., who reported a high rate of treatment dissatisfaction and discontinuation due to limited awareness and unrealistic expectations about pharmacological options [9]. Notably, surgical or device-based interventions were not reported in our sample, potentially indicating low access to or awareness of such modalities in the primary care context. In the Middle East and North Africa, there is a gap in culturally appropriate and accurate data regarding men’s sexual health, as conversation around sexuality is considered taboo or impolite [29]. A recent study reported that among Saudi laypersons, stigma plays a significant role in hindering the process of seeking psychological help for mental illness. However, the item on “Loss of sexual interest or pleasure” was removed from the Arabic version of Hopkins Symptom Checklist-25, aimed to measure anxiety and depression, as it was considered culturally inappropriate [30]. Thus, the perception was prevalent across social groups including healthcare professionals themselves. Most patients who have type 2 diabetes are not asked about ED within the last year of attendance, even though most are willing to discuss it with their physicians. A cross-sectional survey among Saudi men with type 2 diabetes revealed that older age and having more severe ED deterred patients from discussing their sexual health with their physicians. Moreover, even though most patients were willing to discuss it with their physicians, they were not asked about ED by the physicians within the last year of attendance [31]. Despite the availability of effective pharmacological treatments, including PDE5Is, our findings highlight substantial gaps in their utilization. This underutilization may stem from social stigmas, inadequate health literacy, and prevalent misconceptions regarding ED treatment options [32]. Furthermore, evidence suggests that a holistic approach to ED management, incorporating medical, psychological, and social interventions, is essential for optimizing patient outcomes [32,33]. Despite the high burden of sexual dysfunctions on their quality of life, men in the Middle East have limited access, low engagement, and low completion rates for treatment for sexual dysfunctions. Strategies for treatment should consider the sociocultural factors that influence treatment-seeking and engagement behaviors necessary for successful outcomes.

Although the necessity of seeking treatment was acknowledged by half of the participants, the social stigma and a preference for self-medication inhibited half of the participants from reaching out to specialized clinics. The observed underutilization of ED healthcare services in our study is consistent with international evidence, suggesting that stigma and cultural barriers significantly influence help-seeking behavior [9]. Moreover, the lack of health education, limited access to sexual health services, and digital misinformation contribute to further reluctance in seeking professional help. Misinformation, particularly from unreliable online sources, often leads to misconceptions about ED and its treatment options, delaying appropriate care.

To address these challenges, comprehensive strategies must be implemented. Public health initiatives should prioritize accessible, evidence-based education tailored to the sociocultural context. In parallel, there is an urgent need to strengthen the role of healthcare providers by equipping them with the knowledge and communication skills necessary to initiate discussions about sexual health. In conservative settings such as the Middle East, where conversations around sexual function are often avoided, clinician-led dialogue is especially critical. Previous studies have highlighted that reluctance among healthcare providers to address sexual concerns significantly hinders early diagnosis and appropriate management of ED [12,31]. Integrating structured training on sexual health into both undergraduate medical education and continuing professional development programs may improve clinician confidence, normalize patient–provider communication, and ultimately reduce the burden of untreated sexual dysfunction.

### Limitations

The reliance on self-reported data may introduce response biases, potentially affecting the accuracy of findings. Additionally, the cross-sectional design of this study limits the ability to establish causal relationships between erectile dysfunction and associated factors. Furthermore, the generalizability of findings to other populations may be restricted due to the cultural specificity of the study sample. Additionally, body mass index (BMI) was not measured in this study, which limits the ability to assess the relationship between obesity and erectile dysfunction. Moreover, the potential influence of spousal or partner involvement on treatment-seeking behavior was not examined and represents a meaningful area for future investigation.

## 5. Conclusions

In conclusion, this study highlights the prevalence and management challenges of ED in the western region of Saudi Arabia, demonstrating substantial gaps between symptom burden and healthcare utilization. The findings emphasize the urgent need for culturally tailored public health initiatives to improve awareness, healthcare accessibility, and treatment engagement for ED in this population.

## Figures and Tables

**Table 1:** Sociodemographic characteristics, weight, exercise, and smoking status among participants (*n* = 384).

Parameter		*n*	Percentage (%)
Age group (Mean ± SD = 39 ± 8.5)	≤34	109	28.4
	35–45	105	27.3
	45–55	90	23.4
	>55	80	20.8
Number of children (Mean ± SD = 3 ± 2)	0	29	7.6
	1	50	13.0
	2	92	24.0
	3	82	21.4
	4	69	18.0
	>4	62	16.1
Number of wives	1	287	74.7
	2	74	19.3
	3	20	5.2
	4	3	0.8
Education level	No formal education	18	4.7
	Primary	22	5.7
	Intermediate	59	15.4
	Secondary	129	33.6
	University	156	40.6
Chronic diseases	Hypertension	74	19.3
	Diabetes mellitus	119	31.0
	Hypercholesterolemia	54	14.1
	Cardiovascular disease (e.g., stroke, angina)	23	6.0
	Kidney failure	5	1.3
	Mental illness	4	1.0
	Neurological disorders	3	0.7
	None	108	28.2
Weight (kg) (Mean ± SD = 86.2 ± 15.7)	≤70	74	19.3
	71–85	123	32.0
	86–100	97	25.3
	>100	90	23.4
Height (cm) (Mean ± SD = 164.3 ± 8.9)	≤155	73	19.0
	156–165	146	38.0
	166–175	107	27.9
	>175	58	15.1
Exercise frequency (per week)	None	139	36.2
	Once	102	26.6
	2–3 times	78	20.3
	4–5 times	38	9.9
	≥6 times	27	7.0
Smoking status	Cigarette smoker	89	23.2
	Hookah smoker	148	38.5
	Electronic cigarette user	61	15.9
	Non-smoker	86	22.4

**Table 2:** Participants’ attitudes towards ED over the past 4 weeks (*n* = 384).

Parameter		*n*	Percentage (%)
Frequency of achieving an erection during sexual activity	No sexual activity	4	1.0
	Almost always or always	116	30.2
	Very often (much more than half the time)	129	33.6
	Sometimes (about half the time)	88	22.9
	Infrequently (much less than half the time)	39	10.2
	Almost never or never	8	2.1
Frequency of having an erection strong enough for vaginal penetration	No sexual arousal	5	1.3
	Almost always or always	124	32.3
	Very often (much more than half the time)	110	28.6
	Sometimes (about half the time)	98	25.5
	Rarely (much less than half the time)	35	9.1
	Almost never or never	12	3.1
Frequency of successful penetration during intercourse	I have never attempted intercourse	4	1.0
	Almost always or always	118	30.7
	Often (much more than half the time)	118	30.7
	Sometimes (about half the time)	93	24.2
	Rarely (much less than half the time)	42	10.9
	Almost never or never	9	2.3
Frequency of maintaining an erection after penetration	I have never attempted intercourse	4	1.0
	Almost always or always	124	32.3
	Often (much more than half the time)	108	28.1
	Sometimes (about half the time)	94	24.5
	Rarely (much less than half the time)	40	10.4
	Almost never or never	14	3.6
Difficulty in maintaining an erection to complete intercourse	I have never attempted intercourse	4	1.0
	Very difficult	2	0.5
	Difficult	54	14.1
	Somewhat difficult	120	31.3
	Mild difficulty	127	33.1
	No difficulty	77	20.1
Confidence in achieving and maintaining an erection	Very high	45	11.7
	High	126	32.8
	Medium	115	29.9
	Low	82	21.4
	Very low	16	4.2

**Table 3:** Participants’ knowledge and awareness regarding ED treatment (*n* = 384), # the percentages in this table refer to the total number of responses (*n* = 176), not the total number of respondents.

Parameter		*n*	Percentage (%)
Sources of information about ED (Multiple responses allowed) (*n* = 176) #	Friends	36	20.4
	Wife	54	30.7
	Doctor	54	30.7
	Pharmacist	28	15.9
	Social media	42	23.9
Perceived need for ED treatment	Yes	190	49.5
	No	194	50.5
Consultation at male clinics for ED	Yes	180	46.9
	No	204	53.1
Reasons for not seeking medical consultation (Multiple responses allowed) (*n* = 235)	ED is not a serious condition	30	12.8
	Reluctant to discuss ED due to social embarrassment	70	29.8
	Not interested in sexual intercourse	100	26.0
	Concerned about the harmful effects of medications	61	26.0
	Satisfied with self-medication	73	31.0
	Medications are available without a prescription	40	17.0
Use of medication for ED	Yes	181	47.1
	No	203	52.9
Preferred treatment modality (Multiple responses allowed) (*n* = 187)	Medical treatment	70	37.4
	Alternative treatments	64	34.3
	Self-treatment	53	28.3
Sources of treatment information (*n* = 186) (Multiple responses allowed) *	Friends	32	17.2
	Wife	39	21.0
	Doctor	52	28.0
	Pharmacist	45	24.2
	Social media	29	15.6
Perceived effectiveness of ED treatment (*n* = 191)	No improvement	29	15.2
	Slight improvement	46	24.1
	Uncertain	57	29.8
	Moderate improvement	35	18.3
	Significant improvement	24	12.5
Perceptions of sexual enhancers (Viagra, Snafi)	Safe	114	29.7
	Unsafe	158	41.1
	Uncertain	112	29.2
Reasons for considering sexual enhancers unsafe (Multiple responses allowed) (*n* = 268) *	Risk of addiction or dependence	69	25.7
	Serious side effects	79	29.5
	Potential cardiovascular risks	74	27.6
	Uncertain	69	25.7

* Results may overlap.

**Table 4:** Association between taking medication for ED and sociodemographic characteristics.

Parameters		Have You Ever Taken Medication for Erectile Dysfunction?		Total (N = 384)	*p* Value *
		Yes	No		
Age group (years)	≤34	42	67	109	0.019
		23.2%	33.0%	28.4%	
	34–45	45	60	105	
		24.9%	29.6%	27.3%	
	45 to 55	46	44	90	
		25.4%	21.7%	23.4%	
	>55	48	32	80	
		26.5%	15.8%	20.8%	
Number of children	0	10	19	29	0.081
		5.5%	9.4%	7.6%	
	1	21	29	50	
		11.6%	14.3%	13.0%	
	2	40	52	92	
		22.1%	25.6%	24.0%	
	3	36	46	82	
		19.9%	22.7%	21.4%	
	4	43	26	69	
		23.8%	12.8%	18.0%	
	>4	31	31	62	
		17.1%	15.3%	16.1%	
Number of wives	1	129	158	287	0.282
		71.3%	77.8%	74.7%	
	2	37	37	74	
		20.4%	18.2%	19.3%	
	3	13	7	20	
		7.2%	3.4%	5.2%	
	4	2	1	3	
		1.1%	0.5%	0.8%	
Educational level	No formal education	10	8	18	0.127
		5.5%	3.9%	4.7%	
	Primary	13	9	22	
		7.2%	4.4%	5.7%	
	Intermediate	33	26	59	
		18.2%	12.8%	15.4%	
	Secondary	63	66	129	
		34.8%	32.5%	33.6%	
	University	62	94	156	
		34.3%	46.3%	40.6%	
Diabetes or hypertension	No	84	110	194	0.128
		46.4%	54.2%	50.5%	
	Yes	97	93	190	
		53.6%	45.8%	49.5%	
Smoking status	Cigarette smoker	39	50	89	0.865
		21.5%	24.6%	23.2%	
	Hookah smoker	70	78	148	
		38.7%	38.4%	38.5%	
	Electronic cigarette user	31	30	61	
		17.1%	14.8%	15.9%	
	Non-smoker	41	45	86	
		22.7%	22.2%	22.4%	

* *p*-value was considered statistically significant if ≤0.05.

**Table 5:** Association between consulting male clinics for ED and sociodemographic characteristics.

Parameters		Have You Consulted Male Clinics Regarding Erectile Dysfunction?		Total (N = 384)	*p* Value *
		Yes	No		
Age	≤34	54	55	109	0.235
		30.0%	27.0%	28.4%	
	34–45	44	61	105	
		24.4%	29.9%	27.3%	
	45–55	38	52	90	
		21.1%	25.5%	23.4%	
	>55	44	36	80	
		24.4%	17.6%	20.8%	
Number of children	0	13	16	29	0.394
		7.2%	7.8%	7.6%	
	1	23	27	50	
		12.8%	13.2%	13.0%	
	2	35	57	92	
		19.4%	27.9%	24.0%	
	3	41	41	82	
		22.8%	20.1%	21.4%	
	4	38	31	69	
		21.1%	15.2%	18.0%	
	>4	30	32	62	
		16.7%	15.7%	16.1%	
Number of wives	1	135	152	287	0.198
		75.0%	74.5%	74.7%	
	2	31	43	74	
		17.2%	21.1%	19.3%	
	3	11	9	20	
		6.1%	4.4%	5.2%	
	4	3	0	3	
		1.7%	0.0%	0.8%	
Educational level	Uneducated	13	5	18	0.270
		7.2%	2.5%	4.7%	
	Primary	11	11	22	
		6.1%	5.4%	5.7%	
	Intermediate	26	33	59	
		14.4%	16.2%	15.4%	
	Secondary	60	69	129	
		33.3%	33.8%	33.6%	
	University	70	86	156	
		38.9%	42.2%	40.6%	
Diabetes or hypertension	No	86	108	194	0.313
		47.8%	52.9%	50.5%	
	Yes	94	96	190	
		52.2%	47.1%	49.5%	
Smoking status	Cigarette smoker	38	51	89	0.554
		21.1%	25.0%	23.2%	
	Hookah smoker	76	72	148	
		42.2%	35.3%	38.5%	
	Electronic cigarette user	28	33	61	
		15.6%	16.2%	15.9%	
	Non-smoker	38	48	86	
		21.1%	23.5%	22.4%	

* *p*-value was considered statistically significant if ≤0.05.

## Data Availability

The data are available and can be provided upon request.
